# The intergenerational transmission of suicidal behavior: an offspring of siblings study

**DOI:** 10.1038/s41398-020-0850-6

**Published:** 2020-05-30

**Authors:** Lauren M. O’Reilly, Ralf Kuja-Halkola, Martin E. Rickert, Quetzal A. Class, Henrik Larsson, Paul Lichtenstein, Brian M. D’Onofrio

**Affiliations:** 1 Indiana University, Bloomington, IN USA; 2 Karolinska Institutet, Solna, Sweden; 3 University of Illinois, Chicago, IL USA; 4 Örebro University, Örebro, Sweden

**Keywords:** Clinical genetics, Human behaviour

## Abstract

We examined the extent to which genetic factors shared across generations, measured covariates, and environmental factors associated with parental suicidal behavior (suicide attempt or suicide) account for the association between parental and offspring suicidal behavior. We used a Swedish cohort of 2,762,883 offspring born 1973–2001. We conducted two sets of analyses with offspring of half- and full-siblings: (1) quantitative behavior genetic models analyzing maternal suicidal behavior and (2) fixed-effects Cox proportional hazard models analyzing maternal and paternal suicidal behavior. The analyses also adjusted for numerous measured covariates (e.g., parental severe mental illness). Quantitative behavior genetic analyses found that 29.2% (95% confidence interval [CI], 5.29, 53.12%) of the intergenerational association was due to environmental factors associated with exposure to maternal suicidal behavior, with the remainder due to genetic factors. Statistical adjustment for parental behavioral health problems partially attenuated the environmental association; however, the results were no longer statistically significant. Cox hazard models similarly found that offspring were at a 2.74-fold increased risk [95% CI, 2.67, 2.83]) of suicidal behavior if their mothers attempted/died by suicide. After adjustment for familial factors and measured covariates, associations attenuated but remained elevated for offspring of discordant half-siblings (HR, 1.57 [95% CI, 1.45, 1.71]) and full-siblings (HR, 1.62 [95% CI, 1.57, 1.67]). Cox hazard models demonstrated a similar pattern between paternal and offspring suicidal behavior. This study found that the intergenerational transmission of suicidal behavior is largely due to shared genetic factors, as well as factors associated with parental behavioral health problems and environmental factors associated with parental suicidal behavior.

## Introduction

Research has consistently suggested that offspring of suicidal parents are at greater risk for suicidal behavior themselves^1,2^. A recent meta-analysis concluded that family history of self-injurious behaviors was moderately associated with offspring suicide attempt (odds ratio [OR], 1.57)^3^. However, it is unclear *how* the risk of family history of suicidal behavior is transmitted^2–4^. Researchers have proposed potential causal mechanisms including contagion^5–9^ and exposure to adverse environments^10–19^. Parents also share genetic makeup with their offspring; consequently, the association between parental and offspring suicidal behavior may be confounded by genetic factors (i.e., passive gene-environment correlation)^20^. Twin, family, and adoption studies have consistently indicated that suicidal behavior is heritable^7,21–24^. The comorbidity between psychopathology and suicidal behavior^25^ also suggests that parental behavioral health problems may confound the association. Stated differently, the transmission of suicidal behavior between parent and offspring may not be specific to the exposure of parental suicidal behavior, but explained by behavioral health problems (e.g., being raised by a parent with psychopathology may result in a chaotic home environment), which is a common risk factor for suicidality^26^.

Although previous studies have statistically adjusted for measured covariates (e.g., parental psychiatric disorder), attempts to draw causal inferences about the intergenerational association have been limited due to the inability to rigorously adjust for unmeasured genetic and environmental factors^26–28^. To date, adoption studies have been the primary research design used to account for unmeasured factors. While the results from these studies support the role of genetic influences on suicidal behavior^9,29,30^, adoption studies have several limitations (e.g., matching adoptees to families of higher socioeconomic status)^31^ and have not formally examined the intergenerational transmission of suicidal behavior. Therefore, more genetically informed research is needed to assess the processes through which suicidal behavior is transmitted from parents to offspring.

The primary aim of this study was to examine the processes accounting for the intergenerational transmission of suicidal behavior through systematically ruling out non-causal processes. To do so, we first estimated the extent to which genetic and environmental factors account for the intergenerational transmission of suicidal behavior using quantitative behavior genetic modeling of offspring of half- and full-siblings. Given that half- and full-cousins share approximately 6.25% or 12.5% of their segregating alleles, respectively, quantitative behavior genetic modeling can estimate the degree to which common genetic and environmental factors specific to the exposure of parental suicidal behavior account for the association^32^. Second, we used fixed-effects Cox regression models to further compare differentially exposed cousins (i.e., pairs in which one cousin experienced parental suicidal behavior and the other did not), which account for unmeasured familial factors when examining a specific risk (i.e., parental suicidal behavior). Through this comparison and the inclusion of measured covariates, we sought to differentiate among several processes that co-occur in traditional epidemiological studies^33^.

## Materials and methods

### Data

The Internal Review Board at Indiana University and the Regional Ethical Review Board in Stockholm, Sweden, approved this study. We obtained data for the current study from eight national Swedish registers. The Medical Birth Register records nearly all pregnancies in Sweden beginning in 1973^34^. We linked all cohort members to their parents and grandparents using the Multi-Generation Register, which includes familial relations among individuals born after 1932 or living in Sweden since 1961^35^. We identified parental twin pairs from the Swedish Twin Register, which includes nearly all twin pairs born in Sweden from 1886 through 2000^36^. The National Patient Register includes inpatient hospital records since 1964 with complete coverage beginning in 1987 based on International Classification of Diseases (*ICD*) codes (Supplementary Table 1)^37^. The Cause of Death Register includes details on more than 99% of deaths beginning in 1961^38^. The Swedish Migration Register records both immigration and emigration dates for individuals since 1969^38^. The National Crime Register contains criminal conviction data for all individuals over the age of 15 since 1973^39^. Finally, the Education Register includes all information regarding highest level of educational attainment since 1990^40^.

### Sample

#### Cohort

The cohort consisted of 2,891,267 offspring born 1973–2001. We excluded individuals who died (*n* = 21,287 [0.7%]) or emigrated (89,579 [3.1%]) before age 12. Given that our primary research interest was the transmission of parental suicidal behavior to offspring, we excluded all offspring if the date of their first suicidal behavior occurred before their mother’s (*n* = 1,146 [<0.1%]) or father’s (844 [<0.1%]) first recorded suicidal behavior. We also excluded offspring missing maternal or paternal country of origin (*n* = 27 [0.0%] and 15,600 [0.5%], respectively). In total, we excluded 4.44% (*n* = 128,384) of the available cohort, to obtain the final cohort of 2,762,883 unique offspring born to 1,445,546 mothers and 1,449,162 fathers.

#### Exposure and outcome

We derived all information about parental and offspring suicide attempt and death by suicide using data from the National Patient Register and Cause of Death Register, respectively. We included both intentional and undetermined intent self-injurious behaviors to define suicidal behavior, consistent with previous research^22^. Information about International Classification of Disease codes used for identification can be found in Supplementary Table 1. We defined suicidal behavior for both generations as the first recorded suicide attempt requiring inpatient hospitalization or death by suicide after age 12, as the reliability of suicidal behavior before is unclear^41^. Prior research using Swedish registers has documented that childhood and adolescence are high-risk periods for suicidal behavior after exposure to parental suicidal behavior^11,42^; therefore, we restricted exposure to parental suicidal behavior prior to age 18, including parents whose first suicidal behavior occurred before offspring birth.

#### Covariates

We considered offspring parity (first, second, third, or fourth or higher) and maternal and paternal age at offspring birth (in seven groups) as offspring-specific covariates. Maternal- and paternal-specific covariates were highest level of educational attainment (in six groups and a missing category), being born in Sweden, severe mental illness (i.e., lifetime history of either schizophrenia spectrum disorder or bipolar disorder after the age of 12 as recorded in the National Patient Register), and criminal conviction after the age of 15. While certain variables may be more theoretically intuitive as covariates (e.g., parental mental illness), the decision to include these variables was based on prior research^11,41,43,44^, their associations with parental and offspring suicidal behavior (Supplementary Table 2), and the likelihood that these variables temporally preceded the exposure and outcome^43,44^. Of note, offspring parity, parental age at childbearing, parental country of origin, and parental educational attainment served as demographic factors and/or as proxies for socioeconomic status, which may be related to parental suicidal behavior through processes such as chaotic home environment, lack of financial resources, and poor decision-making. We included maternal and paternal covariates to help account for environmental factors that differed within the cousin pair and potential confounding due to assortative mating^45^.

#### Identification of cousin pairs

We identified all sibling pairs within the parent generation and then subsequently determined offspring of siblings (i.e., cousin pairs). Among offspring of sister-sister, brother-brother, or sister-brother parents, we identified all cousin pairs based on those with the same maternal grandmother identifiers, paternal grandmother identifiers, or maternal grandmother and paternal grandmother, respectively. For offspring-of-full-sibling analyses, we excluded offspring of dizygotic (DZ) and monozygotic (MZ) twins identified either from the Swedish Twin Register^46^ or as opposite-sex individuals born on the same day (*n* = 9861 unique cousin pairs). However, we included these individuals in an offspring-of-twins sensitivity analysis. If offspring were missing grandmother and grandfather identifiers, they did not contribute to the analyses.

#### Analyses

To help specify the processes underlying the intergenerational transmission, we conducted two sets of analyses in which we fit a series of: (1) quantitative behavior genetic models and (2) Cox proportional hazard models. Both approaches estimated the association between parental and offspring suicidal behavior and addressed limitations that were inherent to one another. Access to code is available upon request.

#### Quantitative behavior genetic models

**Fig. 1:**
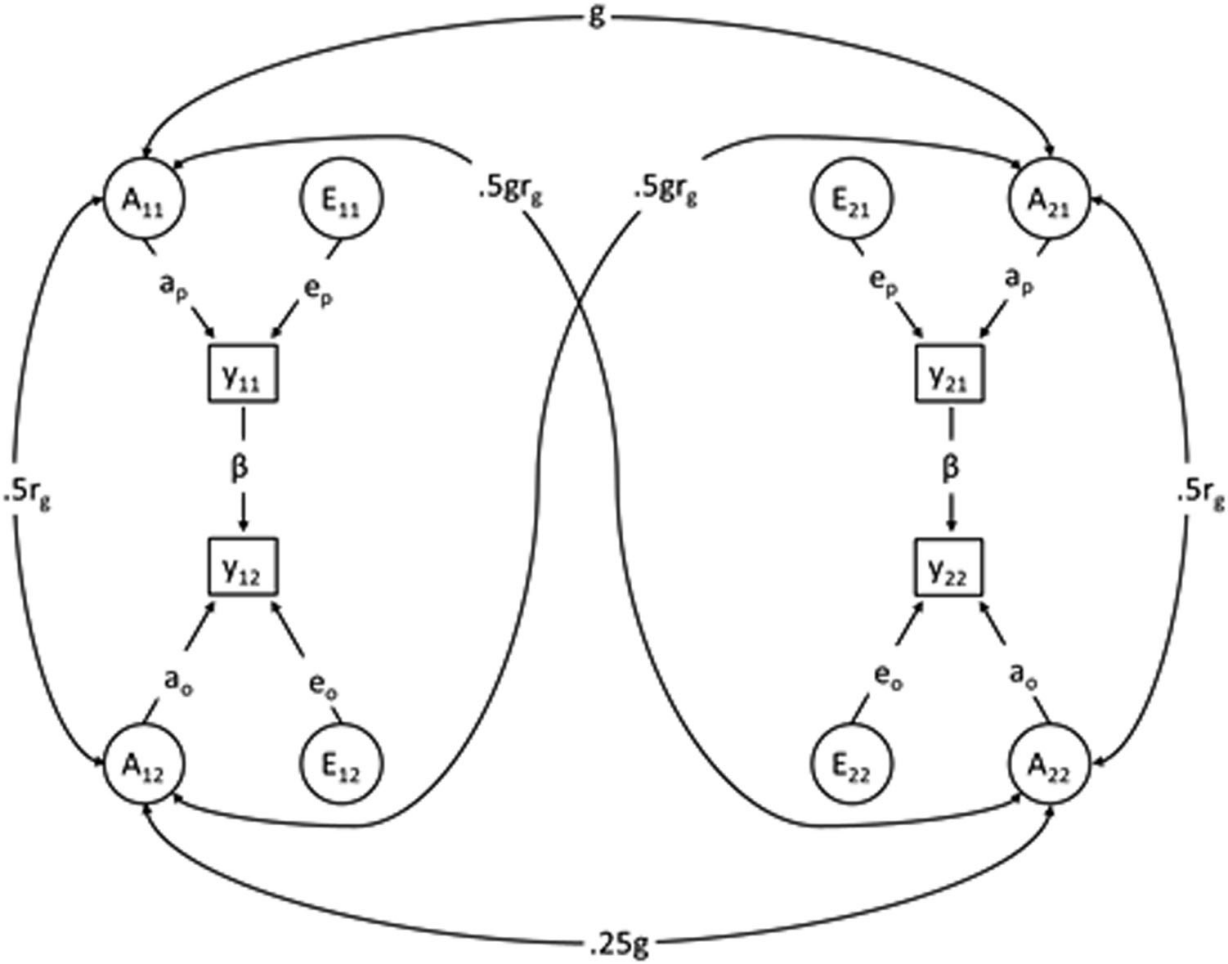
A simplified path diagram for the structural equation model. Note: Represents one example family with each parent having one offspring; the full model includes up to two offspring per parent. The inclusion of both offspring of half-sibling and full-sibling parents allowed us to decompose the variance of genetic (A) and environmental (E) factors. Contribution of shared environment (C) are also estimated, but left out of the figure. Y_11_ and Y_21_ represent two siblings in the parent generation. Y_12_ and Y_22_ represent two cousins in the offspring generation; A_11_ and A_21_ represent the parental additive genetic sources of variance, and g represents the genetic similarity between the two (i.e., 0.50 for full-siblings and 0.25 for maternal half-siblings); E_11_ and E_21_ represent the unique environmental contribution to the variance in the parental phenotype. A_12_ and A_22_ represent the offspring additive genetic sources of variance, and 0.25g represents the genetic similarity between the two; E_12_ and E_22_ represent the unique environmental contribution to the variance in offspring phenotype; r_g_ is the genetic correlation between the parental and offspring phenotype, thus 0.50r_g_ is the correlation between the parental and offspring phenotype due to shared genetics; similarly, 0.50gr_g_ is the correlation between uncle/aunt and niece/nephew due to shared genetics. Parental and offspring may have different proportion of variance explained by A and E, as seen by having different path coefficients (e.g., a_p_ and a_o_). Finally, the direct, phenotypic intergenerational association is modeled by β, where the variance in parental phenotype, regardless of source, may directly influence the variance in offspring phenotype. A description of the model can be found in Supplementary Appendix 1 and in Kuja-Halkola et al.^32^.

First, in order to formally estimate the extent to which the intergenerational association was due to genetic and environmental factors, we fit structural equation models that decomposed the variance of parental and offspring suicidal behavior into additive genetic (A), shared environmental (C; environmental factors that make individuals similar), and nonshared environmental (E; environmental factors that make individuals dissimilar and measurement error) factors. We derived A, C, and E factors for both the parents and offspring separately, which we were able to estimate through the comparison of half- and full-siblings in both generations (see Fig. 1 for a simplified representation of the quantitative behavior genetic models). Of note, A, C, and E are modeled additively in explaining the variance for the observed parental and offspring suicidal behavior. We constrained the correlations among these latent factors across individuals based on genetic relatedness. As such, we assumed the correlation between genetic factors across a parent and offspring to be approximately 50%. The avuncular genetic correlation (between offspring and aunt/uncle) was half of the parent-sibling genetic correlation. The models also included a direct phenotypic path from parental to offspring suicidal behavior in order to capture the intergenerational association that was not explained by the genetic correlation between parental and offspring suicidal behavior. The implemented models were an extension of methods used by Kuja-Halkola et al.^32^, in which the liability towards suicidal behavior was assumed to follow a normal distribution. In this liability-threshold model, we estimated the associations between liabilities using the dichotomous observations of parental and offspring suicidal behavior. For a mathematical description of the models and an analytic solution to the quantitative behavior genetic models, see Supplementary Appendices 1 and 2. We included up to two offspring of each parent who were either half- or full-siblings. We then only included same-sex parent siblings and randomly removed repeated extended families in order to eliminate the dependency between families (rows). See Supplementary Fig. 1 for examples of the types of extended families included in the quantitative behavior genetic analyses. We restricted the quantitative behavior genetic analyses to estimate the processes associated with *maternal* suicidal behavior for two reasons. First, half-siblings are more likely to live with their mothers and thus be exposed to their suicidal behavior, compared to paternal half-siblings. Second, the modeling assumes that the differences in the half- and full-sibling correlations in the offspring generation are due to genetic differences and shared effects of parental suicidal behavior. Paternal half-sibling correlations were not in line with this assumption (Supplementary Table 3).

We fit three models, which included sequential covariate adjustment. First, we fit the quantitative behavior genetic models while only adjusting for parent-sibling and offspring-sibling type (i.e., half- or full-siblings) and differences in the expected prevalence of parental and offspring suicidal behavior. Second, in order to account for the role of comorbid maternal behavioral health problems and offspring characteristics, we adjusted for propensity scores associated with both. We calculated a propensity score from the covariates for mothers and offspring indicating the probability of suicidal behavior using a logistic regression model. For the creation of maternal propensity scores, we included educational attainment, country of origin, severe mental illness, substance use, and criminal convictions. For the creation of offspring propensity scores, we included offspring year of birth, parity, and maternal age at childbearing. Third, to capture potential bias due to assortative mating, we included the paternal propensity scores, in addition to the maternal and offspring propensity scores. We fit all models in a structural equation framework using OpenMx^47^.

#### Cox proportional regression models

In order to relax some of the assumptions of behavior genetic models and increase sample size, we used Cox proportional hazard models to estimate the within-pair (i.e., fixed-effects) estimate among offspring of half- and full-siblings. We also examined associations with both maternal and paternal suicidal behavior, as the sibling correlations among the offspring generation (i.e., the comparison of half- versus full-siblings) did not influence our estimates of the intergenerational association.

We first compared individuals to unrelated individuals in the general population and in the subsets of children of half- and full-siblings. We then assigned a unique identifier to each cousin pair in the sample and stratified on this identifier to obtain fixed-effect estimates, which adjusted for all factors shared within cousin pairs. We accounted for offspring represented in more than one cousin pair by using clustered standard errors^48^. Cousin pairs that contributed to the estimate were those who were discordant on both exposure and outcome (Supplementary Table 4). The models accounted for right censoring of offspring follow-up time; if offspring did not have suicidal behavior within the follow-up period, they contributed to person-time at risk until death, emigration, or end of study date (December 31, 2013), whichever occurred first. For both the population and fixed-effects models, we also included a set of offspring and parental covariates. We conducted the general population analyses in SAS 9.4 and fixed-effects analyses in Stata 13.1^49^.

#### Sensitivity analyses

We performed several sensitivity analyses to address potential bias in our results due to methodological decisions or our dataset. The quantitative behavior genetic models assumed that the genetic correlation between mother and offspring was freely estimated and the heritability of maternal and offspring suicidal behavior were not constrained to be equal. In order to test these assumptions and compare model fit, we examined the estimates when modifying model constraints (i.e., constraining the genetic correlation between mothers and offspring to be either 0 or 1, and when constraining the heritability between parents and offspring to equivalent). As mentioned previously, we conducted the Cox hazard models among offspring of DZ and MZ twins to examine whether the pattern of results held in a sample who shared more environmental (e.g., in utero) and/or genetic factors (e.g., parental twin pairs share either 100% or, on average, 50% of their segregating alleles).

## Results

Table 1 summarizes the cohort demographics, including details for both offspring- and parent-specific variables. Table 2 summarizes Kaplan–Meier estimates of offspring suicidal behavior at age 30.

**Table 1:** Frequency of offspring- and parent-specific variables from cohort of offspring born 1973–2001 (*n* = 2,762,883).

Offspring-specific variables^a^	*N* (%)^b^	
Female	1,343,788 (48.64)	
Parity		
First^c^	1,138,244 (41.20)	
Second	1,018,225 (36.85)	
Third	430,299 (15.57)	
Fourth or higher	176,115 (6.37)	

	**Maternal*N*(%)**^b^	**Paternal*N*(%)**
Age at childbearing		
≤19	98,966 (3.58)	22,084 (0.80)
20–24	639,205 (23.14)	338,894 (12.27)
25–29^c^	1,023,453 (37.04)	901,707 (32.64)
30–34	698,158 (25.27)	852,102 (30.84)
35–39	257,495 (9.32)	427,374 (15.47)
40–44	43,945 (1.59)	153,448 (5.55)
≥45	1659 (0.06)	67,274 (2.43)
Offspring exposed to parental suicidal behavior	87,358 (3.16)	77,671 (2.81)
<Age 18	71,071 (2.57)	60,931 (2.21)
≥Age 18	16,287 (0.59)	16,740 (0.61)
Offspring with suicidal behavior and parents with suicidal behavior^a^	5428 (0.20)	4525 (0.16)

Parent-specific variables^a^	Maternal *N* (%)^b^	Paternal *N* (%)^b^
Born in Sweden	2,437,258 (88.21)	2,417,714 (87.51)
Highest educational attainment		
Primary/lower (<9 years)	111,069 (4.02)	215,096 (7.79)
Primary/lower (9 years)	263,487 (9.54)	364,571 (13.20)
Upper/secondary (1–2 years)^c^	946,236 (34.25)	968,155 (35.04)
Upper/secondary (3 years)	421,867 (15.27)	387,744 (14.03)
Post-secondary (<3 years)	411,654 (14.90)	353,520 (12.80)
Post-secondary (≥3 years)/post-graduate	597,392 (21.62)	443,188 (16.04)
No information/missing	11,178 (0.40)	30,609 (1.11)
Bipolar^d^	16,802 (0.61)	11,722 (0.42)
Schizophrenia^d^	5,912 (0.21)	5,549 (0.20)
Non-substance induced psychosis^d^	24,600 (0.89)	19,161 (0.69)
Substance use^d^	70,563 (2.55)	140,229 (5.08)
Criminal convictions^d^	340,405 (12.32)	1,129,050 (40.86)
Suicide attempt	83,505 (3.02)	62,894 (2.28)
Suicide	7185 (0.26)	19,731 (0.71)
Suicidal behavior	87,358 (3.16)	77,671 (2.81)

^a^Based on 2,762,883 unique offspring.

^b^Percentages rounded to the nearest hundredths and thus may not equal 100.

^c^Reference group.

^d^Lifetime occurrence.

**Table 2:** Kaplan–Meier estimates of offspring suicidal behavior.

Offspring suicidal behavior	Kaplan–Meier Estimates	
	Offspring with suicidal behavior (*N*)	Proportion of suicidal behavior (*N*/10,000 people)

	By age 30	
Suicide attempt^a^	69,161	359
Suicide^a^	3263	19.6
Suicidal behavior^a^	71,777	382

Note: suicidal behavior is defined as either suicide attempt or death by suicide. Given that individuals can both attempt and later die by suicide, the sum of suicide attempt and suicide is less than the frequency of suicidal behavior.

^a^Occurring ≥ age 12.

Maternal and offspring suicidal behavior were correlated (tetrachoric correlation=0.15, confidence interval [CI], 0.13, 0.17]). The quantitative behavior genetic analysis found that 29.2% (95% CI, 5.29, 53.12%) of the association was due to environmental factors specific to exposure to maternal suicidal behavior, whereas the remainder of the association was due to genetic factors shared across the generations (Table 3). When adjusting for offspring and maternal and then adding paternal propensity scores, the association due to specific environmental factors was attenuated and became statistically nonsignificant to 20.7% (95% CI, −19.29, 60.68%) and 15.7% (95% CI, −20.19, 51.57%), respectively. In addition to attenuating the association between parental and offspring suicidal behavior due to environmental factors, the inclusion of propensity scores attenuated the heritability and elevated nonshared environmental influences on parental suicidal behavior (from 0.50 to 0.31, and 0.50 to 0.69, respectively), suggesting genetic factors that influenced the covariates accounted for a proportion of the genetic factors influencing parental suicidal behavior.

**Table 3:** Structural equation model estimates of the processes underlying the association between maternal and offspring suicidal behavior.

	$\hat \beta$ (95% CI)	$\widehat {r_g}$ (95% CI)	$\widehat {a_p^2}$ (95% CI)	$\widehat {c_p^2}$ (95% CI)	$\widehat {e_p^2}$ (95% CI)	$\widehat {a_o^2}$ (95% CI)	$\widehat {c_o^2}$ (95% CI)	$\widehat {e_p^2}$ (95% CI)	Covariance due to $\beta$ (95% CI)
Model with minimal adjustment^a^	0.04 (0.001, 0.08)	0.39 (0.27, 0.51)	0.50 (0.46, 0.54)	0.00	0.50 (0.46, 0.54)	0.61 (0.57, 0.65)	0.00	0.39 (0.35, 0.43)	29.2% (5.29, 53.11%)
Model with adjustment for offspring and maternal propensity score^b^	0.02 (−0.02, 0.06)	0.31 (0.17, 0.45)	0.33 (0.27, 0.39)	0.00	0.67 (0.61, 0.73)	0.60 (0.56, 0.64)	0.00	0.40 (0.36, 0.44)	20.7% (−19.28, 60.68%)
Model with adjustment for offspring, maternal, and paternal propensity score^c^	0.01 (−0.03, 0.05)	0.30 (0.18, 0.42)	0.31 (0.25, 0.37)	0.00	0.69 (0.63, 0.75)	0.60 (0.56, 0.64)	0.00	0.40 (0.36, 0.44)	15.7% (−20.17, 51.57%)

Note: *β* represents the direct path from parent to offspring, *r*_*g*_ represents the genetic correlation between parent and offspring, $a_p^2$ represents the heritability of parental suicidal behavior, $c_p^2$ represents the shared environmental influence on parental suicidal behavior, $e_p^2$ represents the nonshared environmental influence on parental suicidal behavior, $a_o^2$ represents the heritability of offspring suicidal behavior, $c_o^2$ represents the shared environmental influence on offspring suicidal behavior, and $e_p^2$ represents the nonshared environmental influence on offspring suicidal behavior. The covariance due to *β* captures the covariation between parent and offspring suicidal behavior not due to genetic and environmental confounding (i.e., due to the direct effect). The model assumes that *β* and *r*_*g*_ are freely estimated and the heritability of parental and offspring suicidal behavior are different.

^a^Includes adjustment for parent-sibling and offspring-sibling type and differences in the expected prevalence of parental and offspring suicidal behavior.

^b^Additionally includes adjustment for offspring (derived from year of birth, parity, and parental age at childbearing) and maternal propensity score (derived from educational attainment, country of origin, severe mental illness, substance use, and criminal convictions).

^c^Additionally includes adjustment for paternal propensity score (derived from educational attainment, country of origin, severe mental illness, substance use, and criminal convictions).

When estimating the intergenerational association using Cox proportional hazard modeling, individuals exposed to maternal suicidal behavior (hazard ratio [HR], 2.74 [95% CI, 2.67, 2.83]) were at elevated risk for their own suicidal behavior compared to unrelated, unexposed individuals in the general population (Table 4), even after adjusting for the measured covariates (HR, 1.75 [95% CI, 1.69, 1.81]). The magnitude of the intergenerational association was lower when comparing differentially exposed cousins. In the maternal fixed-effects models without statistical adjustment, offspring of half-siblings (HR, 1.94 [95% CI, 1.81, 2.09]) and full-siblings (HR, 1.98 [95% CI, 1.92, 2.04]) were at an increased risk of suicidal behavior compared to their unexposed cousin, though the magnitudes of the associations were attenuated compared to the general population estimate. The estimated risk of suicidal behavior further attenuated when additionally adjusting for measured covariates (HR, 1.57 [95% CI, 1.45, 1.71] and HR, 1.62 [95% CI, 1.57, 1.67], respectively for offspring of half-sibling and full-siblings). Associations with paternal suicidal behavior demonstrated a similar pattern of results, although risk for offspring suicidal behavior was greater if exposed to maternal suicidal behavior compared to paternal. In the general population without adjustment for covariates, offspring were at an elevated risk for suicidal behavior after exposure to paternal suicidal behavior (HR, 2.45 [95% CI, 2.38, 2.53]), although the adjustment for covariates attenuated the association (HR, 1.54 [95% CI, 1.49, 1.59]). Among the population of offspring of half-siblings and full-siblings, the risk was lower (HR, 1.79 [95% CI, 1.66, 1.93] and HR, 1.71 [95% CI, 1.66, 1.77], respectively) compared to the general population. When adjusting for factors shared by differentially exposed offspring of half- and full-siblings, the risks were further attenuated (HR, 1.35 [95% CI, 1.24, 1.47] and HR, 1.38 [95% CI, 1.34, 1.43], respectively) (Table 4).

**Table 4:** Hazard rate of suicidal behavior in the offspring generation among offspring exposed to parental suicidal behavior in different comparison groups.

	Offspring suicidal behavior HR (95% CI)					
Comparison Group	Offspring of unexposed parent from the general population^a^		Offspring of unexposed half-siblings^b^		Offspring of unexposed full-siblings^c^	
	Unadjusted	Adjusted^d^	Unadjusted	Adjusted^d^	Unadjusted	Adjusted^d^
Maternal suicidal behavior						
General population	2.74 (2.67, 2.83)	1.75 (1.69, 1.81)	2.27 (2.10, 2.45)	1.56 (1.42, 1.70)	2.74 (2.62, 2.85)	1.74 (1.66, 1.83)
Cousin-pair comparison	–	–	1.94 (1.81, 2.09)	1.57 (1.45, 1.71)	1.98 (1.92, 2.04)	1.62 (1.57, 1.67)
Paternal suicidal behavior						
General population	2.45 (2.38, 2.53)	1.54 (1.49, 1.59)	2.08 (1.92, 2.26)	1.40 (1.28, 1.53)	2.46 (2.35, 2.58)	1.54 (1.47, 1.62)
Cousin-pair comparison	–	–	1.79 (1.66, 1.93)	1.35 (1.24, 1.47)	1.71 (1.66, 1.77)	1.38 (1.34, 1.43)

Note: Includes offspring who were exposed to parental suicidal behavior before age 18; parental suicidal behavior is either maternal or paternal suicidal behavior. The general population models were conducted in SAS 9.4, and the cousin comparison models were conducted in Stata 13.1.

^a^Based on 2,762,883 unique offspring.

^b^Based on 316,910 unique offspring.

^c^Based on 2,207,801 unique offspring. Note that children of twins are excluded from this sample.

^d^Adjustment includes offspring parity, and parental age of offspring birth, highest level of educational attainment, being born in Sweden, severe mental illness (i.e., schizophrenia spectrum disorder or bipolar disorder), and criminal conviction.

*Sensitivity analyses*

When testing different assumptions in the quantitative behavior genetic modeling via model constraints, the model included in the main analyses conferred the best model fit and most reasonable interpretation (Supplementary Table 5a, b). Thus, our results supported that parent and offspring phenotypes were different, heritability in the parent- and offspring-generation differed, and the genetic correlation between suicidal behavior in the two generations were less than unity. Children of DZ and MZ twin analyses also yielded complementary results as the main analyses, though the confidence intervals around the estimates were quite large (Supplementary Table 6). Specifically, in the general population, offspring exposed to maternal suicidal behavior were at a two-fold increased risk for suicidal behavior (HR, 2.05 [95% CI, 1.50–2.79]), which then attenuated when adjusting for covariates (HR, 1.40 [95% CI, 0.99–1.99]). When comparing cousins exposed to maternal suicidal behavior, offspring were at a 50% increased risk without covariate adjustment (HR, 1.51 [95% CI, 1.11–2.13]), which attenuated slightly when further adjusting for covariates (HR, 1.41 [95% CI, 0.96–2.06]).

## Discussion

When accounting for genetic factors and comorbid parental behavioral health problems, the intergenerational association between parental and offspring suicidal behavior persisted, albeit attenuated from the association identified in the general population. Taken together, the results suggest that: (1) genetic factors cannot completely explain the intergenerational association, although they account for roughly 70% the association; (2) measured covariates account for a portion of the association, above and beyond shared genetic factors; and (3) the remaining (approximately 15%) association is due to environmental factors specifically associated with parental suicidal behavior, potentially suggesting a non-genetic, independent intergenerational association.

The heritability of suicidal behavior has been well-replicated by adoption, twin, and family studies^7,22,29,50^, which is consistent with our quantitative behavior genetic findings that genetic factors largely account for the intergenerational association. Suicidal ideation and behavior are highly comorbid with psychiatric problems, and, as such, comorbid parental behavioral health problems may confer increased risk for offspring suicidal behavior through both genetic and environmental processes^22^. We found that when we included parental propensity scores, the heritability of parental suicidal behavior attenuated due to the shared genetic overlap with other behavioral health problems.

When adjusting for parental propensity scores, the transmission of psychopathology did not entirely explain the transmission of suicidal behavior, which is consistent with both our Cox proportional hazard results and previous literature examining the intergenerational transmission of anxiety, neuroticism, and depression^21,51–56^. The remaining environmental mediation suggests that having a parent who displayed suicidal behavior may confer an increased risk for offspring suicidal behavior through mechanisms such as contagion^5–9^, bereavement after parental loss^10–12^, negative parenting style (e.g., hostility)^22,57^, or chaotic home environment^13–15^. In addition to the interpretation of a direct environmental effect, there may be two alternative explanations: first, there may be cohort-specific genetic effects for suicidal behavior and second, there may be differing genetic effects on adult versus adolescent suicidal behavior. While we did not stratify our quantitative behavior genetic analyses by birth cohort within the parental and offspring generation, our results did suggest that the additive genetic component of suicidal behavior was not perfectly correlated across generation. This may be due to differing genetic factors influencing the generations. The latter explanation of differing heritability estimates by developmental period has been supported by prior studies that have found that heritability estimates increase over the lifespan for various phenotypes (e.g., alcohol use, smoking, depression, and anxiety)^58,59^ and the genetic influences on behavior differ by age of onset^60,61^, but it is unclear whether these findings apply to suicidal behavior. While outside the scope of the current paper, future research will need to explore these possibilities.

When examining both maternal and paternal suicidal behavior in the Cox proportional hazard models, the magnitude of risk for offspring exposed to maternal suicidal behavior was slightly higher compared to exposure to paternal suicidal behavior. This finding is consistent with other research, which has hypothesized that because mothers are often the primary caregivers, their suicidal behavior has a greater impact on offspring suicidal behavior compared to fathers^14,42^. It is important to note, however, that while maternal suicidal behavior may be a greater risk factor for offspring suicidal behavior, the clinical implications may be similar for paternal and maternal suicidal behavior. Children exposed to parental suicidal behavior continue to be at an elevated risk and require additional clinical attention. We also have insufficient statistical power to examine the interaction between parental and offspring gender and risk for suicidal behavior, although previous research suggests that differences among genders may depend on the developmental period of exposure^62^.

This study advances the field of suicidal behavior in two important ways. First, to the best of our knowledge, this is the only use of the offspring-of-siblings design to the study of the intergenerational transmission of suicidal behavior. This design allowed us to adjust for within-extended-family unmeasured confounding, providing a stronger test of causal inference than prior studies comparing unrelated individuals. Second, we used both quantitative behavior genetic analyses and Cox proportional hazard models with fixed-effects to examine the intergenerational association, which have different strengths and limitations. The quantitative behavior genetic analyses estimated the extent to which the maternal-offspring association was due to maternal exposure while simultaneously adjusting for common genetic factors. However, these models included a restricted sample of families (i.e., with up to two offspring of each parent, and same-sex parental siblings) and did not adjust for right censoring. In contrast, the use of proportional hazard modeling allowed us to adjust for right-censored data, include all possible cousin pairs (e.g., born to parents of opposite sex), and examine paternal suicidal behavior. The ability to both quantify the intergenerational transmission and replicate the pattern of findings in a much larger sample is a significant contribution to the field.

This study also has several limitations. First, an assumption of the offspring-of-siblings design is that offspring of full-siblings are directly comparable to offspring of half-siblings^63^. Given that the unadjusted population estimates from the Cox proportional hazard models in offspring of half-siblings were lower than that in the population estimates in the subset of children of full-siblings, this assumption may be violated^50^. However, the use of the quantitative behavior genetic analyses used a different scale for association (i.e., tetrachoric correlations) and was similar on this scale. Second, the offspring-of-siblings design is unable to adjust for environmental factors unique to each nuclear family^55^ and address assumptions related to assortative mating^64^. We included both maternal and paternal measured covariates to limit this bias, but we cannot make a definitive causal inference. Third, we had limited precision in our estimates for the quantitative behavior genetic models. When including propensity scores as covariates, the estimated direct transmission from parent to offspring was no longer statistically significant, hindering the interpretation of the extent to which the intergenerational transmission was consistent with a causal association. However, the converging results from the Cox proportional hazard models strengthened these findings. Fourth, our quantitative genetic models were linear models; we did not explore genetic-environment interactions, as our primary research aim was to examine the main effect of the intergenerational transmission while rigorously adjusting for unmeasured and measured confounding factors. Gene-environment interactions within the context of the intergenerational transmission of suicidal behavior is an important future research direction. Fifth, we did not adjust for parental depression as we only had inpatient *ICD* codes for depressive disorders, which is likely to be highly correlated with inpatient suicide attempt. However, previous research suggests the intergenerational association persists after accounting for parental depression^65^. Sixth, all records of severe mental illness and suicidal behavior are derived from health care data using *ICD* codes, which likely limits our definition to severe events. Specific to suicide attempt, we included self-injurious behavior due to undetermined intent to account for potential misclassification of suicide attempt; however, we were unable to capture suicide attempts that did not present in a hospital setting. Additionally, the use of lifetime occurrences of suicidal behavior does not account for the risk of suicidal behavior to vary over time^3,25^. Our models did not account for repeated events of parental suicidal behavior, which may confer increased risk for offspring. Future research should examine how offspring risk for suicidal behavior develops after exposure to numerous parental suicide attempts and death by suicide. Future research should also examine suicide attempt and suicide separately, as we were unable to stratify by outcome in the current study. Finally, we examined all offspring suicidal behavior prior to age 18, but did not further investigate narrower age ranges that may be particularly sensitive periods in childhood and adolescence. Previous research suggests that the magnitude of the association between parental and offspring suicidal behavior is greater for childhood exposure compared to adolescence and young adulthood^11,62,66^. Continued genetically informed research is needed to further develop our understanding of developmental periods sensitive to parental suicidal behavior exposure.

## Conclusions

This study found that the intergenerational transmission of suicidal behavior is due to genetic factors shared across the generations and factors associated with comorbid behavioral health problems. A remaining association, however, was due to environmental factors specifically associated with exposure to parental suicidal behavior, consistent with a causal interpretation. Research examining the intergenerational transmission of various disorders should consider using multiple analytic approaches. Future suicidality research that can further specify genetic and environmental processes as well as specific mechanisms underlying the intergenerational transmission will help inform clinical interventions. Importantly, however, future research that examines environmental mediators needs to do so in a genetically informed context, as genetic factors appear to explain a large portion of the intergenerational association between parents and offspring. Without accounting for unmeasured confounding factors, researchers may overestimate the impact of a possible mediator, resulting in potentially weak or ineffective behavioral interventions. As continued genetically informative research is needed to elucidate mechanisms that can help inform interventions among offspring who are bereaved and/or offspring who are experiencing suicidality themselves, we reiterate the call outlined by prior research and organizations for continued systematic screening of suicidality and thorough assessments of family history^67^.

## Supplementary information

Supplemental Material
