# Supplementary material for: The intergenerational transmission of suicidal behavior: an offspring of siblings study
Source: Transl Psychiatry. 2020 May 30;10:173. doi: 10.1038/s41398-020-0850-6 (PMC7261287; doi:10.1038/s41398-020-0850-6)
Supplement: Supplementary file 1 — Supplemental Material [file 41398_2020_850_MOESM1_ESM.docx]

**Supplementary Information**

O’Reilly, L.M., Kuja-Halkola, R., Rickert, M.E., et al. The Intergenerational Transmission of Suicidal Behavior: An Offspring of Siblings Study.

**Supplementary Table 1.** Description of the population and healthcare registers and the *International Classification of Disease (ICD)* codes used to measure offspring and parental suicidal behavior parental severe mental illness.

**Supplementary Table 2.** Associations between covariates and offspring and parental suicidal behavior.

**Supplementary Appendix 1**. Mathematical description of the structural equation models.

**Supplementary Appendix 2**. Analytic solution to behavior genetic models.

**Supplementary Figure 1.** Four examples of extended families that were included in quantitative genetic analyses.

**Supplementary Table 3.** Tetrachoric correlations among pairs within and across parent-offspring generations.

**Supplementary Table 4.** Frequency of exposure discordant/exposure and outcome discordant cousin pairs and Kaplan-Meier estimates.

**Supplementary Table 5a**. Structural equation model estimates of the processes underlying the association between maternal and offspring suicidal behavior when modifying model assumptions.

**Supplementary Table 5b**. Model fit statistics when comparing structural equation models between maternal and offspring suicidal behavior.

**Supplementary Table 6**. Associations between parental suicidal behavior and offspring suicidal behavior among offspring of twins.

| **Supplementary Table 1.** Description of the population and healthcare registers and the *International Classification of Disease (ICD)* codes used to measure offspring and parental suicidal behavior parental severe mental illness. | | | | | |
| --- | --- | --- | --- | --- | --- |
| Outcome | Parental Risk Period Start and End Dates | Offspring Risk Period Start and  End Dates | ICD-8 | ICD-9 | ICD-10 |
| Suicide attempt (parental and offspring) | January 1, 1973-December 31, 2010 | January 1, 1985-December 31, 2013 | E950-E959, E980-E989 | E950-E959, E980-E989 | X60-X84, Y10-Y34, Y870, Y872 |
| Severe mental illness (parental) | January 1, 1973-December 31, 2010 | - | - | - | - |
| Bipolar disorder | - | - | 296.1, 296.3, 296.8 | 296A, 296C-296E, 296W | F30-F31 |
| Schizophrenia spectrum disorders | - | - | 295, 297-299 | 295A-295G, 295W, 295X, 297-298 | F20-F29 |
| Inpatient substance abuse (parental) | January 1, 1973-December 31, 2010 | - | 291, 303, 304 | 303-305  (except 305B) | F10-F19 (except F17) |
| Note: All *ICD* codes derived from the National Patient Register. | |  |  |  |  |

| **Supplementary Table 2.** Associations between covariates and offspring and parental suicidal behavior. | | | |
| --- | --- | --- | --- |
|  | OR (95% CI) | | |
| Offspring-Specific Covariates^1^ | Offspring Suicidal Behavior | Maternal Suicidal Behavior | Paternal Suicidal Behavior |
| Year of birth | 0.96 (0.96-0.96) | 1.00 (1.00-1.00) | 0.98 (0.98-0.98) |
| Female | 1.21 (1.19-1.22) | 0.99 (0.98-1.00) | 1.01 (0.99-1.02) |
| Multiple birth | 0.82 (0.78-0.87) | 0.98 (0.93-1.02) | 0.91 (0.86-0.95) |
| Parity |  |  |  |
| First^2^ | REF | REF | REF |
| Second | 1.04 (1.02-1.06) | 0.93 (0.92-0.95) | 0.87 (0.85-0.88) |
| Third | 1.13 (1.10-1.15) | 1.16 (1.14-1.18) | 0.97 (0.95-0.99) |
| Fourth or higher | 1.37 (1.34-1.41) | 1.72 (1.68-1.76) | 1.36 (1.33-1.40) |
| Maternal age at childbearing |  |  |  |
| ≤19 | 2.05 (1.99-2.11) | 3.10 (3.02-3.18) | 2.91 (2.83-2.99) |
| 20-24 | 1.35 (1.33-1.38) | 1.57 (1.55-1.60) | 1.56 (1.53-1.59) |
| 25-29^2^ | REF | REF | REF |
| 30-34 | 0.88 (0.86-0.90) | 0.91 (0.89-0.93) | 0.91 (0.89-0.93) |
| 35-39 | 0.90 (0.87-0.92) | 1.02 (0.99-1.04) | 1.01 (0.98-1.04) |
| 40-44 | 0.97 (0.91-1.03) | 1.09 (1.03-1.16) | 1.16 (1.09-1.23) |
| ≥45 | 0.69 (0.48-0.98) | 0.83 (0.60-1.14) | 0.60 (0.40-0.89) |
| Paternal age at childbearing |  |  |  |
| ≤19 | 2.09 (1.97-2.21) | 3.09 (2.94-3.24) | 3.22 (3.07-3.39) |
| 20-24 | 1.38 (1.35-1.41) | 1.70 (1.67-1.74) | 1.62 (1.59-1.66) |
| 25-29^2^ | REF | REF | REF |
| 30-34 | 0.87 (0.85-0.89) | 0.83 (0.82-0.85) | 0.87 (0.85-0.88) |
| 35-39 | 0.85 (0.83-0.87) | 0.88 (0.86-0.90) | 0.95 (0.93-0.97) |
| 40-44 | 0.90 (0.87-0.93) | 1.00 (0.97-1.03) | 1.07 (1.04-1.11) |
| ≥45 | 0.98 (0.93-1.03) | 1.26 (1.21-1.32) | 1.20 (1.14-1.25) |
|  |  |  |  |
| Maternal-Specific Covariates^1^ |  |  |  |
| Born in Sweden | 1.02 (1.00-1.05) | 0.71 (0.70-0.72) | 0.94 (0.92-0.96) |
| Highest educational attainment |  |  |  |
| Primary/lower (<9 years) | 1.06 (1.02-1.09) | 0.99 (0.95-1.02) | 1.01 (0.98-1.05) |
| Primary/lower (9 years) | 1.23 (1.20-1.25) | 1.77 (1.73-1.80) | 1.42 (1.39-1.45) |
| Upper/secondary (1-2 years)^2^ | REF | REF | REF |
| Upper/secondary (3 years) | 0.73 (0.71-0.74) | 0.65 (0.64-0.66) | 0.75 (0.74-0.77) |
| Post-secondary (<3 years) | 0.71 (0.70-0.73) | 0.52 (0.51-0.53) | 0.60 (0.59-0.62) |
| Post-secondary (≥3 years)/post-graduate | 0.66 (0.64-0.67) | 0.41 (0.41-0.42) | 0.51 (0.50-0.52) |
| Missing/no information | 1.51 (1.38-1.64) | 5.05 (4.80-5.32) | 1.74 (1.61-1.89) |
| Severe mental illness | 1.96 (1.87-2.05) | 14.49 (14.15-14.83) | 2.26 (2.16-2.36) |
| Substance use | 2.65 (2.57-2.73) | 33.06 (32.50-33.63) | 3.83 (3.73-3.93) |
| Criminal convictions | 1.79 (1.75-1.82) | 4.86 (4.79-4.93) | 2.49 (2.45-2.53) |
|  |  |  |  |
| Paternal-Specific Covariates^1^ |  |  |  |
| Born in Sweden | 0.95 (0.93-0.97) | 0.73 (0.72-0.74) | 0.86 (0.84-0.88) |
| Highest educational attainment |  |  |  |
| Primary/lower (<9 years) | 1.10 (1.07-1.13) | 0.92 (0.89-0.94) | 0.98 (0.96-1.01) |
| Primary/lower (9 years) | 1.14 (1.11-1.16) | 1.15 (1.13-1.17) | 1.29 (1.26-1.31) |
| Upper/secondary (1-2 years)^2^ | REF | REF | REF |
| Upper/secondary (3 years) | 0.75 (0.73-0.77) | 0.74 (0.72-0.76) | 0.58 (0.56-0.59) |
| Post-secondary (<3 years) | 0.70 (0.68-0.72) | 0.60 (0.59-0.62) | 0.43 (0.42-0.44) |
| Post-secondary (≥3 years)/post-graduate | 0.63 (0.62-0.65) | 0.49 (0.48-0.50) | 0.34 (0.33-0.35) |
| Missing/no information | 1.78 (1.70-1.87) | 1.73 (1.64-1.81) | 6.65 (6.45-6.86) |
| Severe mental illness | 1.94 (1.84-2.04) | 2.22 (2.12-2.32) | 13.19 (12.84-13.55) |
| Substance use | 2.38 (2.33-2.44) | 3.20 (3.14-3.26) | 20.28 (19.97-20.60) |
| Criminal convictions | 1.66 (1.64-1.68) | 2.30 (2.27-2.33) | 4.25 (4.18-4.32) |
| Note: All variables are associated with offspring and parental suicidal behavior, except for year of birth, female, and multiple birth. ^1^ Based on 2,762,883 offspring. All suicidal behavior occur ≥ age 12. Note that year of birth, offspring sex, and multiple birth variables were not included in the analyses due to lack of association between parental and offspring suicidal behavior. ^2^ Indicates reference category. | | | |

**Supplementary Appendix 1**. Mathematical description of the structural equation models.

The model, with one offspring per parent (as in **Figure 1**), includes measured variables ($\boldsymbol{y}$) and latent variables ($\boldsymbol{\xi},\boldsymbol{\xi}_{2},\boldsymbol{\zeta}$) according to

$$\boldsymbol{y}=\left[ y_{11} y_{21} y_{12} y_{22} \right]^{T}$$

$$\boldsymbol{\xi}=\left[ A_{11} A_{21} A_{12} A_{22} \right]^{T}$$

$$\boldsymbol{\xi}_{2}=\left[ C_{11} C_{21} C_{12} C_{22} \right]^{T}$$

$$\boldsymbol{\zeta}=\left[ E_{11} E_{21} E_{12} E_{22} \right]^{T}$$

The model is described by the equation:

$$\boldsymbol{y=By+\xi+}\boldsymbol{\xi}_{2}\boldsymbol{+\zeta}$$

where

$$\boldsymbol{B}=\left[ \begin{matrix} \begin{matrix} 0 & 0 \\ 0 & 0 \end{matrix} & \begin{matrix} 0 & 0 \\ 0 & 0 \end{matrix} \\ \begin{matrix} \beta& 0 \\ 0 & \beta\end{matrix} & \begin{matrix} 0 & 0 \\ 0 & 0 \end{matrix} \end{matrix} \right]$$

and

$$Cov\left( \boldsymbol{\xi} \right)=\left[ \begin{matrix} \begin{matrix} a_{p}^{2} & ga_{p}^{2} \\ ga_{p}^{2} & a_{p}^{2} \end{matrix} & \begin{matrix} .5r_{g}a_{p}a_{o} & .5gr_{g}a_{p}a_{o} \\ .5gr_{g}a_{p}a_{o} & .5r_{g}a_{p}a_{o} \end{matrix} \\ \begin{matrix} .5r_{g}a_{p}a_{o} & .5gr_{g}a_{p}a_{o} \\ .5gr_{g}a_{p}a_{o} & .5r_{g}a_{p}a_{o} \end{matrix} & \begin{matrix} a_{o}^{2} & .25ga_{o}^{2} \\ .25ga_{o}^{2} & a_{o}^{2} \end{matrix} \end{matrix} \right]$$

$$Cov\left( \boldsymbol{\xi}_{2} \right)=\left[ \begin{matrix} \begin{matrix} c_{p}^{2} & c_{p}^{2} \\ c_{p}^{2} & c_{p}^{2} \end{matrix} & \begin{matrix} 0 & 0 \\ 0 & 0 \end{matrix} \\ \begin{matrix} 0 & 0 \\ 0 & 0 \end{matrix} & \begin{matrix} c_{o}^{2} & 0 \\ 0 & c_{o}^{2} \end{matrix} \end{matrix} \right]$$

$$Cov\left( \boldsymbol{\zeta} \right)=\left[ \begin{matrix} \begin{matrix} e_{p}^{2} & 0 \\ 0 & e_{p}^{2} \end{matrix} & \begin{matrix} 0 & 0 \\ 0 & 0 \end{matrix} \\ \begin{matrix} 0 & 0 \\ 0 & 0 \end{matrix} & \begin{matrix} e_{o}^{2} & 0 \\ 0 & e_{o}^{2} \end{matrix} \end{matrix} \right]$$

where $g$ is genetic relatedness, $a_{p}^{2}$ is heritability of parental trait, and $a_{o}^{2}$ is heritability of offspring trait (similar sub-indexes for $c$ and $e$). All other covariances are assumed to be zero. Note that, across generations, only $r_{g}$ and $\beta$ contributes to the correlation.

So,

$$\boldsymbol{y=By+\xi+}\boldsymbol{\xi}_{2}\boldsymbol{+\zeta}\Leftrightarrow\boldsymbol{y-By=\xi+}\boldsymbol{\xi}_{2}\boldsymbol{+\zeta\Leftrightarrow}\left( \boldsymbol{I-B} \right)\boldsymbol{y=\xi+}\boldsymbol{\xi}_{2}\boldsymbol{+\zeta\Leftrightarrow}$$

$$\boldsymbol{y=}\left( \boldsymbol{I-B} \right)^{\boldsymbol{-1}}\left( \boldsymbol{\xi+}\boldsymbol{\xi}_{2}\boldsymbol{+\zeta} \right)$$

where $\boldsymbol{I}$ is the identity matrix (ones on diagonal, and zeros off-diagonal) of same dimensions as $\boldsymbol{B}$, thus

$$\left( \boldsymbol{I-B} \right)^{\boldsymbol{-}1}\boldsymbol{=S=}\left[ \begin{matrix} \begin{matrix} 1 & 0 \\ 0 & 1 \end{matrix} & \begin{matrix} 0 & 0 \\ 0 & 0 \end{matrix} \\ \begin{matrix} \beta& 0 \\ 0 & \beta\end{matrix} & \begin{matrix} 1 & 0 \\ 0 & 1 \end{matrix} \end{matrix} \right]$$

the implied covariance matrix is the following:

$$Cov\left( \boldsymbol{y} \right)=Cov\left( \boldsymbol{S}\left( \boldsymbol{\xi+}\boldsymbol{\xi}_{2}\boldsymbol{+\zeta} \right) \right)=\boldsymbol{S}Cov\left( \boldsymbol{\xi+}\boldsymbol{\xi}_{2}\boldsymbol{+\zeta} \right)\boldsymbol{S}^{T}\boldsymbol{=}\left[ \begin{matrix} \begin{matrix} 1 & 0 \\ 0 & 1 \end{matrix} & \begin{matrix} 0 & 0 \\ 0 & 0 \end{matrix} \\ \begin{matrix} \beta& 0 \\ 0 & \beta\end{matrix} & \begin{matrix} 1 & 0 \\ 0 & 1 \end{matrix} \end{matrix} \right]\left[ \begin{matrix} \begin{matrix} a_{p}^{2}+c_{p}^{2}+e_{p}^{2} & ga_{p}^{2}+c_{p}^{2} \\ ga_{p}^{2}+c_{p}^{2} & a_{p}^{2}+c_{p}^{2}+e_{p}^{2} \end{matrix} & \begin{matrix} .5r_{g}a_{p}a_{o} & .5gr_{g}a_{p}a_{o} \\ .5gr_{g}a_{p}a_{o} & .5r_{g}a_{p}a_{o} \end{matrix} \\ \begin{matrix} .5r_{g}a_{p}a_{o} & .5gr_{g}a_{p}a_{o} \\ .5gr_{g}a_{p}a_{o} & .5r_{g}a_{p}a_{o} \end{matrix} & \begin{matrix} a_{o}^{2}+c_{o}^{2}+e_{o}^{2} & .25ga_{o}^{2} \\ .25ga_{o}^{2} & a_{o}^{2}+c_{o}^{2}+e_{o}^{2} \end{matrix} \end{matrix} \right]\left[ \begin{matrix} \begin{matrix} 1 & 0 \\ 0 & 1 \end{matrix} & \begin{matrix} \beta& 0 \\ 0 & \beta\end{matrix} \\ \begin{matrix} 0 & 0 \\ 0 & 0 \end{matrix} & \begin{matrix} 1 & 0 \\ 0 & 1 \end{matrix} \end{matrix} \right]=\left[ \begin{matrix} \begin{matrix} a_{p}^{2}+c_{p}^{2}+e_{p}^{2} & ga_{p}^{2}+c_{p}^{2} \\ ga_{p}^{2}+c_{p}^{2} & a_{p}^{2}+c_{p}^{2}+e_{p}^{2} \end{matrix} & \begin{matrix} \beta\left( a_{p}^{2}+c_{p}^{2}+e_{p}^{2} \right)+.5r_{g}a_{p}a_{o} & \beta\left( ga_{p}^{2}+c_{p}^{2} \right)+.5gr_{g}a_{p}a_{o} \\ \beta\left( ga_{p}^{2}+c_{p}^{2} \right)+.5gr_{g}a_{p}a_{o} & \beta\left( a_{p}^{2}+c_{p}^{2}+e_{p}^{2} \right)+.5r_{g}a_{p}a_{o} \end{matrix} \\ \begin{matrix} \beta\left( a_{p}^{2}+c_{p}^{2}+e_{p}^{2} \right)+.5r_{g}a_{p}a_{o} & \beta\left( ga_{p}^{2}+c_{p}^{2} \right)+.5gr_{g}a_{p}a_{o} \\ \beta\left( ga_{p}^{2}+c_{p}^{2} \right)+.5gr_{g}a_{p}a_{o} & \beta\left( a_{p}^{2}+c_{p}^{2}+e_{p}^{2} \right)+.5r_{g}a_{p}a_{o} \end{matrix} & \begin{matrix} \beta^{2}\left( a_{p}^{2}+c_{p}^{2}+e_{p}^{2} \right)+2\beta.5r_{g}a_{p}a_{o}+a_{o}^{2}+c_{o}^{2}+e_{o}^{2} & \beta^{2}ga_{p}^{2}+2\beta.5gr_{g}a_{p}a_{o}+.25ga_{o}^{2} \\ \beta^{2}ga_{p}^{2}+2\beta.5gr_{g}a_{p}a_{o}+.25ga_{o}^{2} & \beta^{2}\left( a_{p}^{2}+c_{p}^{2}+e_{p}^{2} \right)+2\beta.5r_{g}a_{p}a_{o}+a_{o}^{2}+c_{o}^{2}+e_{o}^{2} \end{matrix} \end{matrix} \right]$$

In addition, offspring generation may consist of two siblings within each nuclear family. Thus, by adding an extra sub-index position, we can incorporate the covariance between siblings as

$$Cov\left( \left[ \begin{matrix} y_{121} \\ y_{122} \end{matrix} \right] \right)=\left[ \begin{matrix} a_{o}^{2}+c_{o}^{2}+e_{o}^{2}+\beta^{2}\left( a_{p}^{2}+c_{p}^{2}+e_{p}^{2} \right)+2\beta.5r_{g}a_{p}a_{o} & g_{o}a_{o}^{2}+c_{o}^{2}{+\beta}^{2}\left( a_{p}^{2}+c_{p}^{2}+e_{p}^{2} \right)+2\beta.5r_{g}a_{p}a_{o} \\ g_{o}a_{o}^{2}+c_{o}^{2}{+\beta}^{2}\left( a_{p}^{2}+c_{p}^{2}+e_{p}^{2} \right)+2\beta.5r_{g}a_{p}a_{o} & a_{o}^{2}+c_{o}^{2}+e_{o}^{2}+\beta^{2}\left( a_{p}^{2}+c_{p}^{2}+e_{p}^{2} \right)+2\beta.5r_{g}a_{p}a_{o} \end{matrix} \right]$$

Here $g_{o}$ represents the genetic similarity between offspring siblings.

**Supplementary Appendix 2**. Analytic solution to the behavior genetic model.

Note: The parental full siblings (*g*=0.5) is known, one offspring per parent is assumed, and no C contributions is assumed. The solution is unique, with six unknowns are found from six known variances and correlations. The known variances are:

Variance of parental phenotype = 1.

Variance of offspring phenotype = 1.

The observed correlations are:

Parental full sibling correlation, $r_{F}$.

Offspring cousin correlation, $r_{cou}$.

Parent-offspring correlation, $r_{PO}$.

Avuncular correlation (niece/nephew – uncle/aunt), $r_{avun}$.

Let

$$K=\frac{r_{PO}-\frac{r_{avun}}{r_{F}}}{\frac{1}{2}\sqrt{2r_{F}}-\frac{1}{4}\sqrt{\frac{2}{r_{F}}}}=,$$

then the solutions are:

$$a_{p}^{2}=2r_{F}.$$

$$e_{p}^{2}=1-2r_{F}.$$

$$\beta=r_{PO}-\frac{1}{2}K\sqrt{2r_{F}}.$$

$$a_{o}^{2}=8\left( r_{cou}-\beta^{2}r_{F}-\frac{1}{2}\beta K\sqrt{2r_{F}} \right).$$

$$r_{g}=\frac{K}{\sqrt{a_{o}^{2}}}.$$

$$e_{o}^{2}=1-a_{o}^{2}-\beta^{2}-\beta r_{g}a_{o}a_{p}.$$

The following equations are known (e.g. from Supplementary Appendix 1):

|  | Equation Number: |
| --- | --- |
| Parental variance  $a_{p}^{2}+e_{p}^{2}=1$ | (1) |
| Parental full sibling correlation  $\frac{1}{2}a_{p}^{2}=r_{F}$ | (2) |
| Offspring variance  $a_{o}^{2}+e_{o}^{2}+\beta^{2}+\beta r_{g}a_{o}a_{p}=1$ | (3) |
| Offspring cousin covariance  $\frac{1}{8}a_{o}^{2}+\frac{1}{2}\beta^{2}a_{p}^{2}+\frac{1}{2}\beta r_{g}a_{p}a_{o}=r_{cou}$ | (4) |
| Parent-offspring correlation  $\beta+\frac{1}{2}r_{g}a_{o}a_{p}=r_{PO}$ | (5) |
| Parent-avuncular correlation  $\frac{1}{2}\beta a_{p}^{2}+\frac{1}{4}r_{g}a_{p}a_{o}=r_{avun}$ | (6) |

Solutions:

|  | Equation Number: |
| --- | --- |
| To solve, first (2) yields  $a_{p}^{2}=2r_{F}.$ | (7) |
| And therefore (1) yields  $e_{p}^{2}=1-2r_{F}.$ | (8) |
| From (5) and (7) we can find  $\beta=r_{PO}-\frac{1}{2}r_{g}a_{o}\sqrt{2r_{F}}.$ | (9) |
| From (6) and (7) we can find  $\beta=\frac{r_{avun}}{r_{F}}-\frac{1}{4}r_{g}a_{o}\sqrt{\frac{2}{r_{F}}}.$ | (10) |
| Taking (9) = (10) yields  $r_{PO}-\frac{1}{2}r_{g}a_{o}\sqrt{2r_{F}}=\frac{r_{avun}}{r_{F}}-\frac{1}{4}r_{g}a_{o}\sqrt{\frac{2}{r_{F}}}$  $\Leftrightarrow$  $\frac{1}{2}r_{g}a_{o}\sqrt{2r_{F}}-\frac{1}{4}r_{g}a_{o}\sqrt{\frac{2}{r_{F}}}=r_{PO}-\frac{r_{avun}}{r_{F}}$  $\Leftrightarrow$  $r_{g}a_{o}\left( \frac{1}{2}\sqrt{2r_{F}}-\frac{1}{4}\sqrt{\frac{2}{r_{F}}} \right)=r_{PO}-\frac{r_{avun}}{r_{F}}$  $\Leftrightarrow$  $r_{g}a_{o}=\frac{r_{PO}-\frac{r_{avun}}{r_{F}}}{\frac{1}{2}\sqrt{2r_{F}}-\frac{1}{4}\sqrt{\frac{2}{r_{F}}}}=K,$  where $K$ is calculable. | (11) |
| From (9) and (11) we get  $\beta=r_{PO}-\frac{1}{2}K\sqrt{2r_{F}}.$ | (12) |
| Taking (4) and entering (7), (11), and (12) yields  $r_{cou}=\frac{1}{8}a_{o}^{2}+\frac{1}{2}\beta^{2}a_{p}^{2}+\frac{1}{2}\beta r_{g}a_{p}a_{o}=\frac{1}{8}a_{o}^{2}+\beta^{2}r_{F}+\frac{1}{2}\beta K\sqrt{2r_{F}}$  $\Leftrightarrow$  $a_{o}^{2}=8\left( r_{cou}-\beta^{2}r_{F}-\frac{1}{2}\beta K\sqrt{2r_{F}} \right).$ | (13) |
| Use (11) and enter (13)  $r_{g}=\frac{K}{\sqrt{a_{o}^{2}}}.$ | (14) |
| Finally, use (3) and enter solutions found in (13), (12), (14), and (7)  $e_{o}^{2}=1-a_{o}^{2}-\beta^{2}-\beta r_{g}a_{o}a_{p}.$ | (15) |

**Supplementary Figure 1.** Four examples of extended families that were included in quantitative genetic analyses.

Depicted families include parental maternal full-siblings (A and C), parental maternal half-siblings (B and D), offspring full-siblings (A and B), and offspring half-siblings (C and D). The four examples below are specific to mothers in the parent generation (II), but we created and analyzed an equivalent dataset for fathers.

| A | B |
| --- | --- |
| C | D |

| **Supplementary Table 3.** Tetrachoric correlations among pairs within and across parent-offspring generations. | | | | | | |
| --- | --- | --- | --- | --- | --- | --- |
| **Within generation (tetrachoric correlation, standard error)** | | | | | | |
|  | Parent generation | | Offspring generation | | | |
|  | Full siblings^1^ | Half siblings | Full siblings | Half siblings | Cousins, parent full siblings | Cousins, parents half siblings |
| Mothers | 0.25 (0.01) | 0.09 (0.03) | 0.30 (0.01) | 0.18 (0.02) | 0.13 (0.01) | 0.06 (0.02) |
| Fathers | 0.24 (0.01) | 0.09 (0.03) | 0.29 (0.01) | 0.09 (0.03) | 0.13 (0.01) | 0.08 (0.03) |
|  |  |  |  |  |  |  |
| **Across generation** | | | | | | |
|  | Parent-offspring, parents full siblings | Parent-niece/nephew, parents full siblings | Parent-offspring, parents half siblings | Parent-niece/nephew, parents half siblings |  |  |
| Mothers | 0.15 (0.01) | 0.07 (0.01) | 0.14 (0.02) | 0.04 (0.02) |  |  |
| Fathers | 0.14 (0.01) | 0.08 (0.01) | 0.17 (0.02) | 0.04 (0.02) |  |  |
| Note: All values presented are tetrachoric correlations and standard errors in the parentheses. ^1^ Excludes twins. | | | | |  |  |

| **Supplementary Table 4.** Frequency of exposure discordant/exposure and outcome discordant cousin pairs and Kaplan-Meier estimates. | | |
| --- | --- | --- |
| **General Population** | Offspring of Half-Siblings | Offspring of Full-Siblings |
| Number of unique cousin pairs | 487,899 | 5,872,971 |
| Number of unique offspring | 316,910 | 2,207,801 |
|  |  |  |
| **Outcome Discordant** |  |  |
| Number of unique cousin pairs | 26,988 | 272,004 |
| Number of unique offspring | 32,659 | 278,810 |
| Kaplan-Meier Estimates (by age 25) |  |  |
| Number of offspring with suicidal behavior | 24,732 | 273,043 |
| Proportion of suicidal behavior (N/1,000 people) | 485.5 | 457.3 |
|  |  |  |
| **Exposure and Outcome Discordant^1^** |  |  |
| Number of unique cousin pairs | 6,335 | 43,954 |
| Number of unique offspring | 8,310 | 52,251 |
| Kaplan-Meier Estimates (by age 25) |  |  |
| Number of offspring with suicidal behavior | 5,799 | 38,398 |
| Proportion of suicidal behavior (N/1,000 people) | 484.7 | 457.2 |
| ^1^ Derived from those who were exposure discordant. | | |

| **Supplementary Table 5a**. Structural equation model estimates of the processes underlying the association between maternal and offspring suicidal behavior when modifying model assumptions. | | | | | | | | | | | | |
| --- | --- | --- | --- | --- | --- | --- | --- | --- | --- | --- | --- | --- |
| Model | $\beta$ | $r_{g}$ | $h^{2}$ Parents vs. Offspring | $\hat{\beta}$  (SE) | $\hat{r_{g}}$  (SE) | $\hat{a_{p}^{2}}$  (SE) | $\hat{c_{p}^{2}}$  (SE) | $\hat{e_{p}^{2}}$  (SE) | $\hat{a_{o}^{2}}$  (SE) | $\hat{c_{o}^{2}}$  (SE) | $\hat{e_{p}^{2}}$  (SE) | Covariance due to $\beta$^a^ |
| **M1**: Different phenotypes in parents and offspring. | Free | Free | Different | 0.04 (0.02) | 0.39 (0.06) | 0.50 (0.02) | 0.00 (0.00) | 0.50 (0.02) | 0.61 (0.02) | 0.00 (0.00) | 0.388 (0.018) | 29.2% (12.2%) |
| **M2**: Same genetics in parent and offspring and different explained variance. | Free | Fixed at 1 | Different | 0.01 (0.02) | 1.00 (NA) | 0.12 (0.03) | 0.17 (0.02) | 0.71 (0.02) | 0.62 (0.02) | 0.00 (0.00) | 0.378 (0.018) | 9.5%  (10.7%) |
| **M3**: Same genetics and same variance explained in parent and offspring generation (i.e., same phenotype). | Free | Fixed at 1 | Same | -0.03 (0.01) | 1.00 (N/A) | 0.38 (0.02) | 0.09 (0.01) | 0.53 (0.02) | 0.38 (0.02) | 0.09 (0.01) | 0.532 (0.017) | -19.4% (8.5%) |
| **M4**: Different genetics but same variance explained in parent and offspring generation. | Free | Free | Same | 0.07 (0.01) | 0.30 (0.03) | 0.57 (0.01) | 0.00 (0.00) | 0.43 (0.01) | 0.57 (0.01) | 0.00 (0.00) | 0.433 (0.014) | 45.5%  (5.8%) |
| **M5**: Entirely different genetics. | Free | Fixed at 0 | Different | 0.15 (0.01) | 0  (N/A) | 0.521 (0.03) | 0.00 (0.00) | 0.48 (0.03) | 0.60 (0.02) | 0.00 (0.00) | 0.406 (0.018) | 100%  (N/A) |
| Note: M1 is the assumed model in Table 3 of the main text. $\beta$ represents the direct path from parent to offspring, $r_{g}$ represents the genetic correlation between parent and offspring, $a_{p}^{2}$ represents the heritability of parental suicidal behavior, $c_{p}^{2}$ represents the shared environmental influence on parental suicidal behavior, $e_{p}^{2}$ represents the nonshared environmental influence on parental suicidal behavior, $a_{o}^{2}$ represents the heritability of offspring suicidal behavior, $c_{o}^{2}$ represents the shared environmental influence on offspring suicidal behavior, and $e_{p}^{2}$ represents the nonshared environmental influence on offspring suicidal behavior.  The covariance due to $\beta$ captures the covariation between parent and offspring suicidal behavior not due to genetic and environmental confounding (i.e., due to the direct effect). | | | | | | | | | | | | |

| Supplementary Table 5b. Model fit statistics when comparing structural equation models between maternal and offspring suicidal behavior. | | | | | | | | |
| --- | --- | --- | --- | --- | --- | --- | --- | --- |
| Base Model | Comparison Model | ep | -2LL | df | AIC | diffLL | diffdf | p |
| M1 | <NA> | 12 | 305966.4 | 1220591 | -2135216 | NA | NA | NA |
| M1 | M2 | 11 | 305986.3 | 1220592 | -2135198 | 19.93578 | 1 | 8.01E-06 |
| M1 | M3 | 9 | 306084.1 | 1220594 | -2135104 | 117.6611 | 3 | 2.46E-25 |
| M1 | M4 | 10 | 305979.5 | 1220593 | -2135206 | 13.11654 | 2 | 1.42E-03 |
| M1 | M5 | 11 | 305988.4 | 1220592 | -2135196 | 22.03336 | 1 | 2.68E-06 |

| **Supplementary Table 6.** Associations between parental suicidal behavior and offspring suicidal behavior among offspring of twins. | | |
| --- | --- | --- |
|  | Offspring Suicidal Behavior  HR (95% CI) | |
| Comparison Group | Offspring of Unexposed Twins^1^ | |
|  | Unadjusted | Adjusted^2^ |
| Maternal Suicidal Behavior |  |  |
| General population | 2.05 (1.50-2.79) | 1.40 (0.99-1.99) |
| Cousin-pair comparison | 1.54 (1.11-2.13) | 1.41 (0.96-2.06) |
|  |  |  |
| Paternal Suicidal Behavior |  |  |
| General population | 2.86 (2.12-3.85) | 2.06 (1.49-2.85) |
| Cousin-pair comparison | 1.98 (1.39-2.82) | 1.94 (1.31-2.89) |
| Note: Includes offspring who were exposed to parental suicidal behavior before age 18; parental suicidal behavior is either maternal or paternal suicidal behavior. ^1^ Based on 90,752 unique offspring. ^2^ Adjustment includes offspring parity, and parental age of offspring birth, highest level of educational attainment, being born in Sweden, severe mental illness (i.e., schizophrenia spectrum disorder or bipolar disorder), and criminal conviction. | | |
